# Seeking to Be Heard: Reflections on the Value of a Partnership Approach to Involving Victims in the Development of Domestic Abuse Policy and Practice

**DOI:** 10.3390/bs15070960

**Published:** 2025-07-15

**Authors:** Laura Hammond, Silvia Fraga Dominguez, Jenny Richards

**Affiliations:** 1 College of Psychology, Birmingham City University, Birmingham B4 7BD, UK; silvia.fragadominguez@bcu.ac.uk; 2 College of Business, Law and Social Sciences, University of Derby, Derby DE1 1DZ, UK; j.richards@derby.ac.uk

**Keywords:** domestic abuse, police response, support services, knowledge-sharing, co-production

## Abstract

This paper outlines the development and delivery of a novel, collaborative, co-production approach to incorporating lived experience in the development of policy and practice in the area of domestic abuse. “SEEKERS” (Sharing Experience, Expertise and Knowledge for Effective Responses and Support) is an initiative which brings together victims and advocates, police, practitioners and researchers as equal partners. It creates opportunities for them to share their experiences, expertise, and knowledge, so that others can learn from these and use this learning in addressing domestic abuse-related issues more effectively. Throughout this paper, we discuss some of the challenges encountered in developing and delivering activities and how these were addressed. Notable benefits of the approach will be highlighted, as indicated by feedback from those involved in a range of capacities, including police and law enforcement practitioners, policy makers, councillors, service providers, support services, victim advocates and survivors of domestic abuse. It is hoped that this paper will contribute to ongoing discussions regarding the ways in which different agencies and stakeholders can work together more effectively and how we can create methods and spaces to support meaningful interaction, collaboration, and co-production with victims.

## A Note to Readers

Throughout this paper, we use the term “victim”, as this is the term most commonly used in criminal justice policy and practice. However, we acknowledge that many of those who have experienced domestic abuse (DA) prefer alternative terms, such as “survivor”.

We also frequently use the word “women” when referring to victims; however, we acknowledge that other gender identities are also represented in victim groups.

Whilst the paper primarily focuses on the police, we would like to emphasise that the partnership and activities also included a broader array of those working with DA in a range of professional capacities.

It is not our intention to cause any offence by way of our use of terminology, and we would like to extend our apologies to anyone who is uncomfortable or unhappy with any of the terms used here.

## 1. Introduction

“SEEKERS” (Sharing Experience, Expertise and Knowledge for Effective Responses and Support) is a project in which DA victims/survivors, advocates, practitioners—including police and law enforcement professionals—and researchers/academics undertake a range of knowledge-exchange and co-creation activities, sharing their diverse experience and expertise using an active participatory and collaborative approach, to help inform the development of more effective responses to DA.

The approach taken is innovative in numerous ways. To our knowledge, it is the first time that victims/survivors, police, and other statutory agencies have worked in partnership in a way in which everyone is allocated equal and shared power relating to governance, decision-making, development, and capacity building. Activities are driven by need, as perceived and articulated by the different perspectives represented, and so are learner-led and outcome-focused, with content and coverage targeted towards addressing key issues and challenges with input from all involved stakeholder groups.

Underpinning all activities is the sharing of expert evidence and opinion, in a manner akin to more traditional consultancy models. However, in this instance, the primary objective of the work is the development of a representative and sustainable Community of Practice in which the diverse range of experience and expertise of all members is acknowledged and respected, and is used to guide reform and deliver lasting, positive change. The overarching aim is to ensure that responses to DA meet the needs of all involved, with a focus on ensuring equitable access to justice for all victims/survivors of DA.

The present paper represents the culmination of our work so far, presenting a summary of our learning as to how lived experience can effectively be incorporated into responses to DA, including both the challenges and benefits of doing so.

## 2. Background

One in three women experience physical and/or sexual violence in their lifetime (WHO, 2025), most often from a partner or family member within a domestic context (hereafter referred to as “DA”).

The World Health Organisation identifies gender-based violence, including DA, as a major gender inequality and an international health priority (WHO, 2025). National and international policies refer to such violence undermining safety, health, well-being, economic potential, and human rights. They also highlight how gender-based violence poses particular challenges for individuals from marginalised or underserved populations, who experience intersecting forms of oppression, such as racism, homophobia, xenophobia, and economic exploitation which affect their safety, well-being, and their ability to access support (see, for example, The White House, 2021).

The need for increased research efforts focused on addressing gender-based violence has been highlighted, to better understand root causes, create innovative models, and increase data collection and analysis to evaluate promising practices, including identifying and supporting approaches led by and focused on diverse populations. The need for fair, equitable, and inclusive justice systems has also been emphasised, with reference to the need to increase women’s access to justice (The Domestic Abuse Act, 2021). To accomplish this, it is suggested that partners and allies must work together to encourage reform of discriminatory standards across justice systems, including biases in law enforcement.

For a long time now, the need for, and value of, collaborative efforts and responses to DA between the criminal justice system, social service agencies, researchers, schools, public health organisations, and private organisations has been noted; see, for example, the U.S. Violence Against Women Act (VAWA). The UK Government’s guidance for services relating to Violence Against Women and Girls (VAWG) published in 2021 states that “good commissioning should begin with an understanding that VAWG survivors are experts in their own lives and are integral to the design of services” (Home Office, 2021). Victims’ perceptions and experiences are crucial to understanding how effective policing policies and practices are likely to be, and the extent to which they are likely to achieve their objectives (Keeling et al., 2015). A report into Operation Soteria, a programme designed to transform the investigation of rape,^1^ suggested that victim voices must be at the heart of any force improvement plan alongside transparency and feedback (Stanko, 2022).

The UK College of Policing’s Competency and Values Framework (CVF) necessitates that police be inclusive, enabling, and visionary. This requires collaborative engagement with external partners where shared and collective aims are achieved, with co-production in research being part of the continuing nuancing of evidence-based policing approaches (Davies, 2021). However, as things currently stand, appropriate mechanisms for facilitating such collaborations have yet to be established, with effective multi-agency, multi-perspective partnerships—including ones involving victims—still lacking in the sphere of DA (Home Office, 2025).

### 2.1. The Importance of Victim Input in the Development of Policy and Practice

Despite sustained calls for victims to be heard within the Criminal Justice System (e.g., Hague et al., 2003), alongside growing recognition that listening to their voices is essential to meeting their needs and ensuring more effective responses (Shearson, 2021), their narratives are conspicuous by their absence (Brooks-Hay, 2020). Victim perspectives are still not routinely incorporated into policy and practice developments (Ragavan et al., 2018), despite collaboration and co-production being core elements of the continuing nuancing of evidence-based approaches (Davies, 2021).

Traditionally, women who have experienced DA are deemed “hard to reach” in practice, policy, and legislation (Hague et al., 2003); however, they may be best described as “hard to hear”, given the lack of appropriate mechanisms currently in place to enable their voices to be heard. Many women who have experienced DA express a desire to be involved in developing and improving services and feel they should be able to contribute to multi-agency forums and DA services as automatic practice and right (Hague et al., 2003). Further, turning trauma into positive outcomes for those impacted by DA has notable benefits, including psychological health benefits, potential to empower self and others, promotion of meaning-making, mission, communal solidarity, and the larger social/political changes that can emerge from giving voice to silenced experiences (Delker et al., 2020).

As a result of the ongoing lack of consideration of the victim voice, structural inequalities which promote paternalistic, top-down approaches are promulgated (Koss et al., 2017). Alternative approaches are needed which recognise the transformative possibilities of intersectionality, which have a social justice focus, which challenge dominant knowledge paradigms, emphasise the role of interdisciplinarity and reject one-size-fits-all solutions (Warner et al., 2016). Without these, DA victims will continue to be unserved, underserved, or ill-served, especially those who are most vulnerable (Koss et al., 2017).

The benefits and value of involving victims/survivors and specialist service providers through co-production have long been both acknowledged (Hague et al., 2003) and championed (e.g., Davies, 2021). The literature describes benefits for all parties engaging in co-production, with “intrinsic value” for individuals through being connected and valued, and an “increased capacity and impact” for services (Warwick-Booth et al., 2022). Co-production approaches improve well-being, reduce stigma, and build people’s agency and skills (Cattaneo & Goodman, 2010).

Co-production approaches have been successfully employed in related areas. For example, Rape Crisis England and Wales has committed to working with survivors through co-production, with stakeholders having shared ownership of the process of design and delivery of new online services, with an emphasis on the importance of lived experience in informing how services can best meet diverse needs. Their model provides a good example of the adoption of a bespoke, flexible, partnership approach focusing on the proactive inclusion of underserved groups (Warwick-Booth et al., 2022).

The Communication Barriers Working Group (2023) suggests that, going forward, police, criminal justice agencies and government departments should be transparent about how they embed learning from engagement with victims/survivors, including those from marginalised groups, to avoid recurring problems of lack of accessibility, awareness, and equal access to safety and support. In addition, they recommend that practitioners should undertake continuous learning and professional development on DA and victims’ experiences, particularly in relation to the criminal justice system. This, they propose, should be designed and facilitated in collaboration with specialist organisations, representing victim and advocate perspectives.

### 2.2. The Importance of Co-Learning and Co-Development Opportunities

Previous research has indicated that factors like knowledge and attitudes, levels of specialised training, organisational practices, government policy as well as departmental and individual interpretation of standards can all play a role in how an officer responds to DA cases (Balenovich et al., 2008; Ballucci et al., 2017; Saxton et al., 2020; Perez Trujillo & Ross, 2008).

It has been suggested that some of the main barriers to effective criminal justice responses to DA relate to police attitudes and associated biases (Richards et al., 2021). Police attitudes are likely to determine the assessment and responses to particular incidents, as well as how officers interact with victims (Maple & Kebbell, 2021). Victims often describe feeling belittled, disrespected, or dismissed throughout the process (Regehr & Alaggia, 2006), which they say can be traumatising and re-victimising (Spencer et al., 2018). This impacts the likelihood of them engaging with criminal justice processes, of redacting allegations or withdrawing their co-operation (Garcia-Jimenez et al., 2018).

DA presents unique challenges in criminal justice contexts, due to both the volume of offences and complexities in the processing of cases (Maple & Kebbell, 2024). DA requires extensive investment from criminal justice professionals, and many report finding DA cases difficult to address, often feeling hopeless and powerless to effect real change in the victims’ lives (Goodman-Delahunty & Corbo Crehan, 2016). Birdsall et al. (2017) discuss how in DA cases the police often must act as both investigators and mediators, ensuring both the victim’s welfare whilst compiling a strong evidential case, which many of those working in such areas highlight as a particular problem.

Maple and Kebbell (2021) conducted interviews with frontline officers regarding their perceptions of domestic and family violence, reporting that DA incidents are often viewed as the most frustrating and difficult calls for police to attend. Many police officers report difficulty in determining the primary aggressor when responding to an incident involving intimate partners (Gover et al., 2011; Johnson, 2007; Stephens & Sinden, 2000). Police can also have difficulty understanding the complexity of DA and struggle to identify abusive behaviour, which can result in officers blaming victims for remaining with the abusive partner (Richards, 2020).

Several studies indicate that police officers are often frustrated by repeat calls for the same victim and cannot understand why women do not leave abusive relationships (e.g., Gover et al., 2011; Horwitz et al., 2011). Further, many police officers also seem to hold the perception that victims are frequently uncooperative (DeJong et al., 2008; Gover et al., 2011; Johnson, 2007; Stephens & Sinden, 2000). 

In an ethnographic investigation of police culture in the United Kingdom, Loftus (2009) found that officers regarded attending minor domestic disputes as burdensome and unimportant. Officers resented the counselling role required of them and felt their efforts were futile because victims would often withdraw complaints and return to a violent partner only to repeat the cycle (Balenovich et al., 2008). These frustrations underline the conflict between empowering a victim to make informed choices and officers’ desire to administer justice where it is warranted (Stephens & Sinden, 2000).

When officers lack the knowledge, understanding, and resources to make complex decisions to meet victims’ needs, they default to investigation procedures and processes rather than connection with victims (Stanko, 2022). This can have negative consequences with regard to victim engagement and satisfaction (Goodman-Delahunty & Corbo Crehan, 2016). Studies show a strong relationship between victims’ satisfaction with police responses and subsequent likelihood of further engagement, including willingness to call them in the future if needed (Apsler et al., 2003; Leisenring, 2012). Johnson (2007) found that victims who viewed officers as sympathetic, who reported that officers discussed options for dealing with the abuse, and who said that officers had solicited their opinions regarding how to address their situations were the most likely to call the police again.

Addressing officers’ attitudes is therefore important; however, whilst DA training programmes currently offered have proven effective in improving police officers’ understanding of DA, they have had limited success in tackling underlying attitudes (Myhill, 2017). Patterson (2011), amongst others, argues that given the complex nature of DA and victims’ unique needs, improving training must be a priority for law enforcement, especially given the high rates of staff turnover and challenging working conditions observed in relation to the policing of DA (Bates & Douglas, 2020). Rich (2019) observes that tailored training is particularly important in policing contexts where cultural and procedural norms conflict with best practice approaches; for example, where there is pressure to keep crime statistics low, and where avoidance of collaboration with outside parties or external partners is commonplace. He discusses, too, how enriched training can assist the provision of a more trauma-informed approach.

Leisenring (2012) suggests that obtaining insight into victims’ thoughts and perceptions may be useful for officers to help them better understand victim behaviour and assist them in dealing with DA incidents. Murphy-Oikonen et al. (2022) discuss how hearing from DA victims could influence the police to respond to their expressed need for compassionate and sensitive consideration and communication; for example, police officers may learn from these accounts that the questions that they ask when responding to a report can increase harm to women if they are not trauma-informed and sensitive.

Taking a more victim-centred or victim-led approach, shifting the focus away from viewing success as simply comprising certain criminal justice outcomes and towards approaches which focus on the needs, wants, and expectations of victims, could help police derive more satisfaction from responding to DA and family violence incidents (Maple & Kebbell, 2021). Leisenring (2012), for example, suggests that by equipping police officers with a better understanding of DA and greater empathy for victims, they may be better able to respond effectively and appropriately to DA incidents.

### 2.3. The Need for Fair, Equitable, and Inclusive Justice Systems

Despite many of the issues and challenges noted so far being widespread, it has been noted that barriers to receiving effective criminal justice responses and access to justice are particularly prevalent for those from minoritised groups (Ragavan et al., 2018). These largely stem from mistrust and fear of discrimination, as well as previous negative experiences (Boggess & Groblewski, 2011). Immigration status adds additional layers of complexity: immigrant survivors can face language barriers, and a lack of culturally specific resources, and those without the right paperwork may risk deportation (Vidales, 2010).

For many years, concerns have been raised about how public bodies, including the police, often fail to comply with their obligations under equality legislation to eliminate discrimination, harassment, and victimisation when interacting with survivors (see, for example, the work of the Communication Barriers Working Group, 2023). The resultant failure to guarantee equal opportunity to engage with formal systems may prevent certain victim groups from accessing justice and safety; something that is a key issue for policing, and one which policy developments such as the Police Race Action Plan have sought to specifically address.

Ragavan et al. (2018) discuss how projects that involve community partners are more likely to develop culturally relevant definitions of DA that truly reflect the lived experiences of racial and ethnic minority women. For this reason, it has been suggested that finding mechanisms through which the voices of women can be incorporated into the development of police and practice is of utmost importance, in order to reduce inequalities and create fair, equitable and inclusive justice systems (Serrata et al., 2017; White et al., 2013). Similarly, Koss et al. (2017) discuss how by creating and utilising knowledge-sharing and educational opportunities and evidencing the value of co-production in developing policy and practice in relation to DA, it might be possible to provide equitable access to justice, both locally and internationally.

A continued effort must keep moving criminal justice professionals, service providers and academics to a place where they are not the ones talking on behalf of survivors and making assumptions about their wants or needs, but where survivors are able to communicate what they want, and their voices are taken into account in driving change and improving systems.

## 3. SEEKERS: Sharing Experience, Expertise and Knowledge for Effective Responses and Support

### 3.1. Development of “SEEKERS”

SEEKERS developed as the result of an event held in January 2023, where we invited police and other criminal justice practitioners to a seminar showcasing findings from a piece of research we had conducted with the charity WAITS (Women Acting In Today’s Society), who specialise in supporting women from minoritised communities. The work explored the experiences and support needs of DA victims with complex, intersecting needs, with the seminar aiming to facilitate discussion around solutions to the issues and barriers identified by victims. Those who had participated in the research were also invited to attend, to see the potential impacts of their voices in helping to implement change.

Prior to the event, one of the women posted a tweet querying whether those with lived experience should be afforded the opportunity to be involved in the development or delivery of research, knowledge-exchange activities or events. This caused us to reflect on our approach and was the catalyst for the development of the SEEKERS project.

The January 2023 event effectively constituted the first stage of the project development. Recognising that merely inviting victims to attend such events was insufficient in terms of acknowledging and valuing the substantial contributions that they might make as “experts” through their lived experience, we changed the planned structure for the day, utilising the opportunity to bring together stakeholders with different forms of professional and personal experience in relation to DA and hold open, frank conversations regarding the issues and challenges faced in relation to DA.

We developed—with WAITS—a set of focused summaries of key issues identified by victims of DA, often on the basis of their own experience(s), which were presented to attendees at the start of the session. These were then used as the basis for a series of round-table discussions, where participants from each of the different stakeholder groups were encouraged to collectively and collaboratively reflect on the issues identified, and to indicate whether they felt there were any further issues in relation to core areas such as police responses, courts and criminal justice processes, service provision, financial support and housing. They were asked to discuss how these issues might potentially be addressed, and what would be needed in order to deliver meaningful and positive change, and/or to improve systems or processes. Each table made notes, which were collected and collated afterwards, and representatives from each of the tables gave a summary of their thoughts and recommendations to the whole group, which then fed into a final discussion session.

There were around 30 attendees at this event. In terms of the capacity within which they attended the event; representation from the different stakeholder groups was roughly equal, with approximately a third being from WAITS (including the women who had taken part in the initial research project), a third being criminal justice professionals or policy makers, and a third being researchers/academics.^2^

The approach taken proved very successful, with many attendees highlighting how beneficial the conversations were in understanding each other’s perspectives. Notably, police and criminal justice professionals said that these conversations were much more impactful than the DA training they usually receive, and that they felt they had learned more from engaging with the women from WAITS than they had in previous professional activities. Victims said that they were glad to be able to share their experiences and provide a voice to others who might not have had such an opportunity, to help change perceptions and potentially improve responses for victims of DA.

What seemed to be pivotal to the success of this format was the provision of a safe and neutral space, where everyone was treated as an equal, and their experience and expertise respectfully acknowledged.

### 3.2. A Participatory Model for Including Domestic Abuse Survivors as Equal Partners in the Development of Policy and Practice

Using the insights and what we learned from this initial event, including feedback regarding how the event ran, benefits and potential areas for improvement received from participants from the different stakeholder groups, we sought to develop a more formal framework for offering co-learning and co-creation opportunities bringing together those with lived experience of DA from a range of perspectives. In doing so, we consulted—both individually and collectively—the different stakeholder groups, to determine how best to conduct the activities, particularly in terms of things like where and when they took place, and the structure and form that they took. By designing and delivering the subsequent knowledge-exchange events and co-learning sessions in partnership with these stakeholders, we sought to ensure that they provided rich opportunities for inclusion, involvement, and engagement.

In developing our approach, we also referred extensively to literature on partnership and co-production approaches and on participatory epistemologies, with a particular focus on victim engagement and trauma-informed practice. This was to ensure that it was suitable for use in the context of DA and with vulnerable participant groups, but also with the aim of creating a model that was sustainable, adaptable, and—most importantly—effective in terms of what we were seeking to achieve.

Utilising a participatory co-production approach in involving DA victims in the development of criminal justice policy and practice recognises that people who use services have knowledge and experience that can help make services better, not only for themselves but for others who need them, which could be any one of us at some time in our lives (Think Local, 2023). Their input can help increase service capacity and engagement, decrease disparities in service access, and lead to more sustainable services (Koss et al., 2017). Further, Ragavan et al. (2018) note that the shared power and transparent relationship building inherent in such an approach can be particularly important for those minoritised backgrounds, including those from racial and ethnic minorities, helping to build trust and rapport with mainstream agencies and formal services. By enabling them to have a voice and agency, they can be empowered to drive systemic change and ensure that such communities are no longer underserved.

Participatory approaches centralise sharing power and collaborative decision-making between community partners and academics (Jumarali et al., 2021), with parity of expertise being especially important in addressing potential power imbalances between professionals and other community members involved (Warwick-Booth et al., 2022). As such, supportive cultures in which people feel secure and have a sense of belonging are important underpinnings of successful co-production (Warwick-Booth et al., 2022).

Jumarali et al. (2021) note that participatory methods can be difficult to employ when working with vulnerable populations, particularly victims of DA who are at increased risk of experiencing abuse-related trauma and have distinct safety-related needs. They provide valuable guidance with regard to how to implement victim-centred, trauma-informed approaches—albeit in the context of conducting research with DA victims. In particular, we found their suggestions regarding ways of promoting open dialogue and critical consciousness, addressing tensions and building trust and authentic relationships particularly useful, and referred to this extensively in developing our approach.

Trauma-informed care is a strengths-based approach that considers the needs of the victim as a whole person. In the context of DA, trauma-informed approaches encompass six key practices: (1) promoting emotional safety for survivors; (2) restoring their choice and control; (3) facilitating connection amongst survivors; (4) supporting and enhancing their methods of coping; (5) responding to survivors’ identities and contexts including systems of oppression and marginalisation; and (6) building upon their strengths (Goodman et al., 2016; Jumarali et al., 2021). Trauma-informed care involves sharing power with victims through collaborative decision-making, as well as developing opportunities for survivors to contribute and cultivate their skills to guide practice (Wilson et al., 2015). Implementing these practices requires creating a safe, welcoming environment in which survivors can share their stories and build authentic relationships with practitioners and with one another (Menschner & Maul, 2016).

We found the work of both Nichols (2013) and Aldridge (2022) helpful in ensuring that the model we were employing was appropriately victim-centred. To do this, it was important to make their unique contexts and experiences a central focus of activities, to ensure that we were flexible and responsive to them and to their needs, and to cultivate opportunities for advocates to learn from survivors (Nichols, 2013). By doing this, as Koss et al. (2017) discuss, we might then use what we learn from listening directly to victims to expand and enhance justice system methods and processes. Ghanbarpour et al. (2018), Goodman et al. (2017) and Jumarali et al. (2021) all note the importance of knowledge exchange and bi-directional learning as a key element of participatory, co-production approaches, particularly in light of the fact that the expertise of community partners is often neglected or dismissed as a result of the structural oppression embedded within traditional engagement and consultancy methods (Ghanbarpour et al., 2018). Thus, truly participatory methods require that opportunities be established for both parties to learn from one another (Goodman et al., 2017).

Underpinning the model that we have developed are Hague et al.’s (2003) principles of effective consultation with DA victims, which suggest that to be meaningful and effective, consultancy and partnership-work needs: (i) sensitive implementation, cognisant of equality and diversity issues; (ii) mechanisms for converting results into real action and policy change; and (iii) appropriate mechanisms for review, implementation, and for keeping contributors apprised of outcomes.

In formalising this participatory model, we also considered several ethical issues and how these might best be addressed. Participation in any activities was entirely voluntary, and participants were free to leave at any time. This was made clear at all times. At no point were participants obligated to indicate the nature of their professional or personal experience—it was their choice as to whether to disclose their individual experience or perspectives. Throughout, it was made clear that discussions would be collated and summarised, and that by participating they were giving permission for their contributions to be included in any summary outputs, but that no individual would be identifiable in any summaries or outputs, and that organisations would only be referred to with explicit permission (to be obtained prior to any dissemination activities). Where different roles, sectors or sources of lived experience are referred to, generic descriptions are used (e.g., “Victim Representative”—noting that this does not necessarily imply victimhood, “Charity Worker” or “Criminal Justice Professional”).

To protect participants, a clear summary of the scope and coverage of the activities is provided in advance, to enable them to make an informed decision as to whether to participate, and to ensure that they are aware of the potential risks of doing so. At the beginning of any activities appropriate content warnings are given. Sources of support are clearly signposted throughout, including a range of different provisions to ensure that they are suitable for any specific requirements. Further, debrief opportunities are provided to participants, including through the different partner organisations.

All activities are held on a university campus, as this was identified as a safe and secure venue, with restricted access and security services available, to ensure that participants—particularly victims—feel comfortable attending. Further, it is a neutral location which gives no indication that those participating have any connection with DA or with the police; it is an open educational establishment with a diverse and representative student population, and so participants are able to attend sessions in the same way that any other learner groups would, and not look (and should not feel) out of place.

The location of campus where the events take place is central and accessible, serviced by a range of public transport options. There are a range of facilities available to cater for any access issues, disability-related needs (e.g., support for deaf or blind learners where required), parenting-related requirements (e.g., breast-feeding facilities and baby changing facilities), and faith-related requirements (including dedicated faith spaces and facilities). There are also both gender-neutral and gender-specific bathroom facilities across the campus. In addition, support services are available 24-7, which can be accessed if needed.

Sessions are run at times that participants will be most likely to be able to attend, taking into account aspects such as refuge curfews, timings associated with childcare commitments or holidays, as well as annual leave trends.

Since January 2023 we have held a number of SEEKERS events, varying in terms of the number of participants and the topics or issues of focus. However, the format and approach has been kept consistent across these activities, with the key focus being on involving all stakeholders as equal partners in all elements of the design, development and running of the events, as well as in the delivery of any mutually agreed outcomes and in monitoring impacts.

## 4. Key Tenets of the “SEEKERS” Approach

Summarised below are what those who have been involved in SEEKERS activities have suggested are the key tenets and most valuable aspects of our approach. In relation to each, examples are given of how and in what ways these have been implemented, and what the key outcomes or learning from each have been. All the quotes in this section and subsequent sections are from participants in SEEKERS events, with the sector or capacity in which they were attending the event indicated.

### 4.1. Seeking to Understand the Issues

“Lived experiences are paramount if effective change is to take place.”

[Charity Worker]

The sessions enable victims to talk about their experiences of reporting DA (or reasons for not reporting) and subsequent responses from statutory services and support providers. They are able to reflect on the extent to which their needs were met, and to suggest areas for improvement, with regard to the quality of interactions and service provision. Police and practitioners are able to discuss the challenges they face with regard to responding to DA and meeting demands. They are also able to discuss current and planned policy and practice reforms, gaining an opportunity to garner feedback and suggestions on these from different stakeholder groups and partner agencies. It has been noted that feedback from victims is particularly valuable in this respect. Victims also report finding it helpful hearing about the issues and challenges in reconciling their own experiences with the reality of criminal justice systems and processes.

### 4.2. Seeking to Understand Different Perspectives and Experiences

“I have learnt how each agency is doing their best to tackle DA but also what issues they run into along the way and how this negatively affects their ability to carry out their job efficiently.”

[Academic]

One aspect of the sessions which has been particularly valued has been the opportunity to ask open and frank questions—and feeling able to do so. For police, understanding reasons for victims not reporting or engaging, or for withdrawing from criminal processes helps them better understand how and in what ways different forms of intervention or support may be utilised to achieve better outcomes (which may not be formal criminal justice outcomes).

This has led to all participants gaining a greater understanding of barriers—perceived and experienced, personal and practical, individual and systemic—to engaging and interacting with the criminal justice system, including those which particularly impact on minoritised communities, and of limitations or inadequacies in services or support, as summarised in Table 1.

In turn, it has provided opportunities for those from different backgrounds and with different experiences—both lived and professional—to offer suggestions as to potential improvements or solutions.

### 4.3. Seeking to Understand Operational Challenges

“This [event] has helped remind me of the importance of my role and what we can work together to achieve.”

[Council Domestic Abuse Officer]

Victims and advocates who have been involved in the SEEKERS activities have said that one of the most valuable elements for them has been obtaining a better understanding of police and criminal justice responses. Often the police do not explain why they are or are not doing things or taking particular courses of action, which means that victims are left feeling like they have not received the service they are entitled to. They also reflected that some may not have a detailed or accurate understanding of what the police or other agencies can or are able to do. Therefore, having the opportunity to discuss their experiences and gain insights from others as to why things were done the way that they were helped bring some closure, and in some cases made them feel more satisfied with what had happened in their particular case.

Victims also noted that the majority of their understanding of police performance, procedures and practice came from their own experiences, which they recognised might not be representative and only part of a much bigger picture, or from secondary sources, including media/social media coverage. They found hearing about what the police were doing to try and improve responses and provide a better service very reassuring, and in feedback they said that they thought this was a very valuable learning opportunity—one which would often be missed. They also appreciated having the opportunity to provide feedback on initiatives or policy/practice changes, as a means of helping the police to ensure that they were fit for purpose and likely to have the best possible outcomes. The police, too, found this element of the discussions immensely helpful.

### 4.4. Seeking to Recognise and Harness Expertise

“It made me think differently about what it means to survivors to be equal partners in work that is about them and for them.”

[Academic]

Many of those who take part in the activities said that they made them think differently about what it means to be an “expert”, and with regard to where they might garner expert insights and knowledge from. Recognising—and valuing—expertise garnered through experience, whether this was lived, personal or professional experience, proved a core feature of the activities and was key in the development of actionable outcomes and effective solutions to issues identified and problems articulated.

### 4.5. Seeking to Empower

“We’ve brought together survivors, advocates and experts to shine a spotlight on the systematic barriers and create meaningful solutions that we expect these sectors to take on board to engage, support and empower women to survive DA.”

[Victim Representative]

The women who participated in activities as “experts by experience” unanimously reported they had found the experience personally rewarding. Many said that it was really meaningful to have something that they perceived as so positive—helping improve systems and practices—come out of their negative experiences. The general sentiment was that if they could prevent someone else from going through what they did, or from experiencing some of the things that they had experienced, then they were very glad to have had the opportunity to share their experience and help drive positive change.

### 4.6. Seeking to Develop Empathy and Understanding

“It was very powerful to hear the voices of women survivors as they are so often neglected.”

[Victim Representative]

From the outset it was noted that empathy is not something that you can easily teach, nor can you directly alter someone’s attitudes or beliefs via simple training. However, by being able to have open, frank conversations—of this nature, and in this manner—those from different backgrounds and with different experiences said that they left the sessions feeling like they understood better the perspectives of others, that they had greater appreciation of the challenges that they faced, and more empathy for them and their situation.

Perhaps most worthy of note, police attendees said that they felt that they would think and respond to DA cases differently in the future, as a consequence of increased awareness of the issues from the victim perspective. This accords with the propositions of Leisenring (2012) and others, regarding the importance of lived experience in changing police attitudes to DA, and—in turn—potentially improving the way in which the police interact with victims.

### 4.7. Seeking to Identify, Caputre, and Extend Good Practice

“Only by working with our third sector partners, support groups from our diverse community, and listening to the reality of survivors… will we be able to reflect on and improve the services that we provide.”

[Criminal Justice Professional]

From all sides, a key outcome from the discussions was the opportunity to identify and harness examples of good practice, and to consider how these might be extended to improve services and support more broadly. It was noted that policy or practice reviews frequently focus on areas for improvement, but there was recognition that there is excellent work taking place, and partners felt that it was important that consideration was given to that, with opportunities to share best practice being provided for all.

### 4.8. Seeking to Develop More Effective Responses and Solutions

“We spend time talking about the problems… but it’s rare that we actually come up with solutions.”

[Criminal Justice System Professional]

Throughout all the activities undertaken as part of the SEEKERS project, the focus has been on not just talking about the problems, but actively seeking feasible and workable solutions to help address shortfalls or limitations with regard to current responses and support in the context of DA.

After each session or activity, a summary report of discussions, detailing the issues or challenges identified and solutions or developments proposed, was circulated to attendees (and other local partners or stakeholders, as appropriate). Action plans have been used to ensure that—as is so often the case—commitments made to instigating or supporting change were upheld, introducing accountability in terms of delivery at both individual and organisational levels. Subsequent sessions start with a review of actions and updates on implementation, to ensure that all involved are kept abreast of developments and improvements, creating an ongoing cycle to ensure positive and lasting change.

## 5. Outcomes and Benefits of SEEKERS

### Key Outcomes of the Work Undertaken and Approach More Generally

Developed together with project partners, presented in Table 2 is a summary of what we believe are the key outcomes of the SEEKERS project and the work undertaken as part of it.

Following activities or events, we asked attendees to provide feedback on their experience, including how much they felt they had learnt, how important they felt it was that lived experience was being used to inform policy and practice developments in the field, and what they felt that they would take away from the event.

The feedback received is summarised below. All 45 responses received to requests for feedback have been incorporated; however, it should be noted that feedback was given anonymously and, given that some individuals may have attended multiple events, it is possible that the number of respondents is lower than this (i.e., some may have provided feedback on more than one occasion).

Participants were asked how much they felt they had learnt about the lived experience of DA victims and the challenges they faced as a result of attending the event. They responded on a scale from 1 to 10, with 1 being the lowest and 10 being the highest. The average response rating was 9.1.

In free text comments provided, respondents mentioned learning about the support available, what different agencies are each doing, and about developments in specific areas of work (e.g., evidence-led prosecutions), which they felt was demonstrative of the importance of working together and learning from one another to improve systems and make “meaningful, systemic change”. Others commented on the fact that they would take what they had learnt and use in various ways, including working with their colleagues to incorporate insights from lived experiences in reviewing and streamlining processes, and in informing development of their (police) education programme(s).

Relatedly, participants were asked to rate the importance of lived experience for informing developments in their line of work, again responding on a scale from 1 (lowest) to 10 (highest). In this instance, the average response rating was 9.5. In their comments, respondents discussed the need to ensure that service users were truly heard and that their ideas, suggestions and recommendations were considered, saying that the individual should be leading the journey, with support and service providers “assisting them”. Others indicated that the insights and knowledge they had acquired as a result of attending would give them more confidence in advocating for survivors.

When asked whether their perception of the positive value of incorporating lived experienced into their practice had changed as a result of attending the event, 78% responded that it had. When then asked the degree to which their perception had changed as a result of attending the event, the average degree of change was ranked as 9 out of 10. Many said that they enjoyed the sharing of “lived and work experience”, and several highlighted the importance of having different voices and types of experience in the room.

When asked to what extent they thought that what they had learnt by attending the event would inform their practice or work in this area, the average response rating provided (again, out of 10) was 8.8. Most respondents said that they would consider or embed lived experience in their line of work; for example, involving lived experience in commissioning decisions, or by inviting those with lived experience to talk to their teams and departments in a professional capacity. A number said they would continue to capture survivor voices and encourage clients to get involved in consultations. One respondent said they would look to incorporate their learning from the event in implementing their lived experience board. Others said that, as a result of the insights afforded through the information shared, they would seek to shift the focus of their activities to solutions, instead of focusing solely on the problems. Further, several others indicated that they appreciated the focus being on identifying practical solutions, highlighting this as one of the key values of the day.

Participants were also asked to provide an indication as to the extent to which they felt that attending the event helped establish new working links or partnerships (or would help establish them in the future). Ratings were provided on a scale of 1 to 10 (lowest to highest), with the average response rating being 8.6. Respondents said that they enjoyed the interaction and networking with other people and agencies, getting to know what other agencies are doing and coming together to find solutions.

Many referred to having enjoyed the interaction element, group discussions, and having the ability to speak to different tables and attendees. Several felt that the table discussions which took part in the latter part of the session that they attended was the most helpful element of the day, allowing them to focus on specific areas and reflect on earlier discussions and issues raised in the first part of the day. One respondent said they enjoyed “seeing different people from different organisations come together to form and make different opinions”. Similarly, another respondent mentioned that they enjoyed the “open conversations and a want from professionals to learn”.

When asked how likely, on a scale of 1 to 10 (lowest to highest) they were to recommend SEEKERS events to others, the average rating given by respondents was 9.3. Numerous comments were made about the willingness of people from different sectors and backgrounds to come together to create change, and about the many individuals who care and have an important role to play. For example, one respondent said that it “opened [their] eyes to see so many people are passionate to help”. Others said that the event had provided a welcome refresher, and that having a reminder of “why they do what they do” had been invaluable.

Participants were given the option to provide a final free text response; many used this opportunity to provide positive comments thanking the organising team (particularly the representatives from WAITS, who had played a key role in running and organising the events), saying that this was “a very well run and informative event”, “excellent and well organised”, and that we should “keep going with this work”. They also said the event was “warm and inviting” and that they were looking forward to future events, with suggestions that such events be held more frequently, covering different aspects of DA. Respondents said it was “a pleasure to be involved”, and that they were grateful for being able to take part.

## 6. Examples of Impact and Legacy

From a policing perspective, by identifying, harnessing, and promoting good practice through direct engagement with service users, policy and practice might be enhanced, which could ultimately lead to better responses to DA cases, through building capacity and utilising resources more effectively.

One example of this is particularly poignant: during a SEEKERS event, one victim described the distressing experience of being taken into temporary accommodation and finding that she did not have with her a number of items which she would need to be able to apply for housing and financial support (such as her passport, ID and other personal documents). She contacted the police and asked them to retrieve these from the property that she shared with her abuser, as she was worried about going there herself because of the risk of harm and that he might follow her to discover where she was. They said that they would not. This left the victim feeling let down by the police, and she could not understand why they would not help.

When she recounted her experience, police attendees were able to respond and explain that they have no legal powers, after the fact, to retrieve items. Victims said that they found it reassuring to know and to understand why the police would not go to retrieve items in such instances, and that it made them think differently about the police response they had received; they wished that the officers that they had dealt with had explained this to them at the time, and said that they would have felt more satisfied with the response if this had been the case.

Further discussion of this issue led to the suggestion that it might be helpful for those who have experienced DA to prepare a list of essential items they found that they needed and/or wished that they had taken with them when being removed from an abusive situation (including items of a high sentimental value), which could be used by officers attending DA incidents; officers could go through this “checklist” with a victim, and give them the prompts, time and space necessary to collate these items and make sure that they have them with them. Based on these discussions, such lists have been co-produced by SEEKERS partners, and this is now being incorporated into policy and practice—including in mandatory training—by local police forces.

This, for us, is an example of a clear and easy “win” from both sides: it helps police provide a better service and reduces the risk of victim dissatisfaction or the possibility of disengagement, as well as the likelihood of additional calls or requests for assistance. It ensures that victims’ needs are prioritised, something that they say they often feel is not the case. Most significantly, it potentially reduces further risk of harm or unnecessary criminal justice interactions.

The second example was identified in discussions at SEEKERS events. Victims reported feeling helpless and powerless throughout the criminal justice process, being given little by way of information or updates about their cases and not being given any agency or choice with regard to how their cases progressed or their ultimate outcomes. For many, this was seen as a key reason for victim withdrawal and/or reluctance to engage with criminal justice systems and processes. It was noted that being kept informed is part of the Code of Practice for Victims of Crime (2013), but many felt that this element of the code was not consistently being upheld.

Potential solutions were posited by partners, including considering alternative ways of contacting victims and of maintaining contact with them. Police are now employing a range of new technologies to assist in responding to DA, including rapid video responses and advocate-present responses, and have revised policies and practices so that communication with the victim and keeping them informed as to what is happening with their case are prioritised. They have used the SEEKERS group as a means of obtaining feedback on these changes, and to gain insights from victims and advocates as to how they can continue to develop and hone responses to ensure that they are effective and inclusive. DA victims, advocates and support services have welcomed the opportunity to consult with regard to such developments, and, in addition, have found it helpful to be party to these developments, being able to share this information with the community more broadly, as a means of reassuring victims and encouraging reporting and engagement.

These changes have also addressed language and communication barriers that were identified in original discussions as reasons why some victims—particularly those from minoritised or immigrant groups—were being prevented from accessing justice and support. Using video response offers opportunities for concurrent translation, and it also means that interpreters can be made available more readily and much faster than was previously the case.

The final example relates to a broad issue identified through all events: victims of DA, and, indeed, many other types of crime, as well as communities more generally, lack confidence in the police and in their ability to deliver positive and satisfactory outcomes. Enhanced, victim-led and victim-focused learning and development opportunities were seen as a good start towards building trust and confidence, as was open and transparent engagement and co-production with a range of partners to ensure that responses cater more effectively to the needs and concerns of those impacted by DA. Adding a degree of accountability, through the use of such frameworks, was also seen as being very valuable.

Local police services and Police and Crime Commissioners have now introduced lived experience consultancy panels (run as part of the SEEKERS partnership activities), DA scrutiny panels and DA Independent Advisory Groups (IAGs), as well as increased representation of lived experience on strategic boards. Members of the SEEKERS team are also leading on partnership work relating to Equality, Diversity and Inclusivity (EDI) and DA across the region. All of this we view as a very positive step forward, recognising that stronger partnerships and working together build trust and help victims feel more confident about accessing and receiving the justice to which they are entitled.

WAITS and other SEEKERS partners are supporting victims in these endeavours—including through the provision of workshops and training to help build confidence in public speaking and in working with diverse groups/partners—to help ensure that they feel that they are able to have a voice, make themselves heard, and to amplify the voices of others when acting as consultants or “experts by experience”.

“I’m glad to see women with lived experience being invited to make valuable contributions and for them to be recognised”

[Academic]

On a more personal note; we ourselves have found that our focus and priorities in our research and engagement activities have shifted considerably as a result of the work that we have been undertaking as part of SEEKERS. It has changed the way in which we, as academics, think about impact and delivering meaningful change, and how better to work in partnership with stakeholders and beneficiaries, substantially altering our plans with regard to research development going forwards. Quotes such as the one presented above suggest that many of the other academics involved in the activities feel similarly.

## 7. Challenges and Limitations

Developing SEEKERS has been a steep learning curve; whilst we referred to research and good practice guidelines in developing the activities undertaken, as well as the broader approach, we did encounter a range of unforeseen challenges. For example, recognising issues around parity of expertise, we wanted to ensure that those taking part in activities as “experts by experience” felt appropriately valued, and they themselves, as well as others, viewed them as equals. We sought to find ways to redress any imbalance wherever we could, but this has proven challenging. The activities undertaken have been supported by our organisations but have not had any external funding to date. We have applied for funding via various avenues, but what we have found is that our approach—which includes costing in expert consultancy payments for victims, at rates equivalent to those of other partners—does not work with many traditional funding formats. Further, responses suggest that whilst the potential of the approach is realised, as there is no precedent and because much of what we do falls into unchartered territory, that it is perhaps viewed as a risky investment.

Indeed, from the onset a significant challenge has been that the approach we are taking with SEEKERS does not neatly fit standard moulds when it comes to academic research. When submitting papers for conferences or to publication outlets there is often no means of including our victim or policing partners as co-authors, and we have encountered several barriers to having them attend and present at academic events with us. Relatedly, what we have also found—both in applying for funding, and in seeking to disseminate our work—is that it is often very hard to effectively capture and showcase impact from work of this nature. Simple metrics do not appropriately demonstrate broader learning and wider societal benefits, including trickle-down benefits or what might be termed “ripple effects” (such as sharing of learning with non-attendees).

It is also important to acknowledge some limitations of our work and feedback presented herein. The feedback was gathered through self-report; even though this was done anonymously, given the nature of the events and respondents’ knowledge that the feedback would be used to evidence impact of these events, there may have been some social desirability effects. It is also important to consider that those taking part in these events may be more motivated to take part, and they may also benefit more as a result. A priority for future events and future work will be to gather more formal evaluation data, which will allow us to more rigorously measure the impact of the events (and any areas that may require improvement or adjustments moving forwards).

Despite these challenges and limitations we are confident that things are changing, and will continue to change. Publication of this paper is a significant step forwards in this respect. As more start to see and appreciate the value of incorporating lived experience in developing policy and practice, we hope that some of what we have been able to achieve with the project, and the insights and learning that we have shared, will provide guidance and support in this respect.

## 8. Next Steps

Our SEEKERS work and associated partnership activities continue to strengthen and grow. We have now held multiple similar events where different people come together to effect change in addressing issues and improving service responses. This has included using a similar format to understand the perceptions and experiences of other groups, such an event exploring ways of improving police engagement with different communities. Discussions and suggestions from the events are feeding directly into police EDI policies and are being used to inform the development of activities in relation to the Police Race Action Plan. Over the coming months we are hosting further collaborative and co-learning events, bringing victims and communities together with criminal justice partners and service providers to co-create solutions to key issues and prevailing challenges noted in a range of different domains.

We have now co-presented our work at several academic conferences and external events. In keeping with the SEEKERS ethos, where it has not been possible for non-academic partners (including victims and advocates) to attend and co-present, we have instead used video so that their voices are present and that they are included, with the emphasis on them as equal partners in the work.

The feedback that we have received from both academic and professional audiences has been overwhelmingly positive regarding the approach we are taking incorporating lived experience into the development of criminal justice policy and practice. As a result, we have had numerous requests to attend future events, as well as for guidance in adopting this model of co-production for use elsewhere.

Future research developments will focus on developing and testing this as an adaptable model of learning and knowledge exchange, as well as on finding new ways to explore longer-term sustainability, legacy and impact. As part of this, we aim to better understand the experiences and outcomes for all of those participating in these activities, both personally and professionally, as we feel that this should be a key focus which guides further developments.

We invite readers to contact us if they would like to be informed of future developments, or if they would like to be involved in this work going forwards.

## 9. Conclusions and Parting Thoughts

“Victim-driven”, “victim-centred”, and “victim-sensitive” have become common buzzwords in the DA domain. However, as Koss et al. (2017) discuss, truly victim-informed services go beyond inviting victims to the conference table as specimens to be analysed (Koss et al., 2017). We need to be creative in our responses to DA. Rather than working in silos, we need to bring people together to find better solutions that address issues from different perspectives.

By creating educational opportunities within supportive, enriched environments, which enable victims, advocates, service providers (including statutory service providers, such the police and criminal justice professionals) and researchers to share their insights, experiences, and knowledge, the SEEKERS model provides a mechanism for the co-production of more effective responses to DA—ones which are better tailored to the needs of victims and which are feasible, deliverable, and sustainable for service providers.

## Figures and Tables

**Table 1:** Key barriers, as identified through collaborative discussion.

Barriers to Reporting and Interacting with the Criminal Justice System and Criminal Justice Professionals	Barriers to Accessing Justice and to Criminal Justice Responses	Barriers to Accessing Other Adequate Support
Language barriers, including a lack of support for deaf survivors Fear of children being taken away Feeling that police officers demonstrate little or no empathy or understanding Perpetrators’ powers and abilities to “play the system” Being interviewed in front of the perpetrator and without feeling safe Formal interviews, including police stations not being viewed as adequate locations for conducting such interviews	Not being given options by police: for example, being given only one option (to take a legal route) Responding officers creating expectations for victims; investigation teams will often fail to meet these expectations Victims not being informed regarding case progression/outcomes. This includes a lack of appropriate updates, including receiving text notifications of cases being “NFA-ed” (and not knowing what this means).	Refuges not always being appropriate or accessible Police not being aware of which support agencies to refer to Time-limited support, which is inappropriate for some victims’ needs. Issues in accessing support, including the inability to self-refer “No recourse to public funds”, and other associated funding/financial challenges

**Table 2:** Key outcomes, as identified through collaborative discussion.

Outcome	
1	Development and testing of an adaptable model of good practice for facilitating knowledge exchange and reciprocal learning, for use in the criminal justice arena and beyond
2	Evidence as to how a multi-perspective, multi-disciplinary collaborative approach can be utilised effectively in tackling pressing social issues
3	An inclusive mechanism for facilitating engagement and building relationships between academics, police and diverse community partners or groups (including those historically considered to be “hard to access”)
4	A means of integrating lived experience into interactive, reflective training for continued learning and professional development
5	Amplification of the voices of marginalised and minoritised victims of DA, ensuring that these are heard and considered in police development and practice responses
6	Precedents for appropriate recognition and recompense when working with diverse expert groups, to ensure effective and equitable partnerships

## Data Availability

The data are not publicly available due to privacy or ethical restriction.

## Notes

1 https://www.npcc.police.uk/our-work/violence-against-women-and-girls/operation-soteria/ (accessed on 9 July 2025).

2 These breakdowns reflect the capacities within which individuals attended the events, but not necessarily their broader experience or expertise. For example, some may have worked in other roles or sectors previously, and some attending in a particular capacity might also have lived or professional experience in another capacity.

## Abbreviations

The following abbreviations are used in this manuscript:

DA	Domestic Abuse
SEEKERS	Sharing Experience, Expertise and Knowledge for Effective Responses and Support
WAITS	Women Acting In Today’s Society
CPD	Continuous Professional Development
EDI	Equality, Diversity and Inclusivity
